# Retrospective Analysis of 2019-nCov-Infected Cases in Dongyang, Southeastern China

**DOI:** 10.1155/2020/7056707

**Published:** 2020-06-29

**Authors:** G. G. Li, Z. Lv, Y. S. Wang, J. F. Li, L. F. Feng, M. F. Wang, B. He, X. L. Pan

**Affiliations:** ^1^Department of Clinical Laboratory, Affiliated Dongyang Hospital of Wenzhou Medical University, Dongyang, Zhejiang, China; ^2^Administrative Department, Affiliated Dongyang Hospital of Wenzhou Medical University, Dongyang, Zhejiang, China; ^3^Medical Department, Affiliated Dongyang Hospital of Wenzhou Medical University, Dongyang, Zhejiang, China; ^4^Department of Respiratory, Affiliated Dongyang Hospital of Wenzhou Medical University, Dongyang, Zhejiang, China; ^5^Department of Biomedical Sciences Laboratory, Affiliated Dongyang Hospital of Wenzhou Medical University, Dongyang, Zhejiang, China; ^6^Infection-Control Department, Affiliated Dongyang Hospital of Wenzhou Medical University, Dongyang, Zhejiang, China

## Abstract

The 2019 novel coronavirus (2019-nCov) has caused increasing number of infected cases globally. This study was performed to analyze information regarding the transmission route and presence of viral nucleic acids on several clinical samples. Confirmed 2019-nCov-infected cases were identified in Dongyang and were treated according to guidelines for the diagnosis of 2019-nCov infection released by the National Health Commission. Information regarding the contacts that the infected people had was collected to determine whether it caused clustered cases. A series of successive nucleic acid examination of feces, oropharyngeal swabs, and sputum was also performed, and the results were analyzed. A total of 19 confirmed cases of 2019-nCov infection were identified in Dongyang, Zhejiang Province, China. Five cases showed severe symptoms, and the remaining ones showed mild manifestations. Ten cases infected from two asymptomatic individuals were clustered into two groups. Among 14 cases with consecutive nucleic acid test results, four patients showed positive results in feces after their negative conversion in oropharyngeal swabs. Asymptomatic individuals with the virus could cause 2019-nCov clustered cases, and the clustered cases may differ from sporadic cases on age and length of hospitalization. In addition, nucleic acids in feces last longer than those in oropharyngeal swabs.

## 1. Background

The 2019 novel coronavirus (2019-nCov) was first reported in December 2019. It causes pneumonia in individuals, with a mortality rate of 2.3% among confirmed cases, based on data from China [1, 2]. The virus originated from wild animals and has the ability to spread among human beings via close contact [3–7]. As on 3 March, the 2019-nCov has caused more than 80,000 confirmed cases in China (http://www.nhc.gov.cn/xcs/yqfkdt/202003/6e02433f7786402d8ea162fb5b80b5a0.shtml). Although more than 50,000 patients have been cured, an increase in the number of cases outside China has been reported, based on the daily situation report released by the World Health Organization (https://www.who.int/emergencies/diseases/novel-coronavirus-2019/situation-reports). The prevention of transmission among human beings still remains a challenge because of the difficulties in avoiding contacts among people.

With most cases being emerged in China, the clinical characteristics of 2019-nCov infection have been analyzed and increasingly understood [8–11]. Therefore, the “Diagnosis and Treatment Scheme of New Coronavirus Infected Pneumonia” guidelines, announced by the National Health Commission (NHC) of China, have been updated to its seventh version (http://www.nhc.gov.cn/yzygj/s7653p/202003/46c9294a7dfe4cef80dc7f5912eb1989.shtml). Suspected individuals, based on clinical symptoms and their travel history, were identified as positive cases once confirmed of the presence of the virus through the nucleic acid detection test and specific anti-virus immunoglobulin assay [12, 13]. However, some cases showed negative results for the nucleic acid detection test, indicating the necessity of combining the diagnosis with computed tomography scans [14–17]. Moreover, emerging results show that results from nucleic acid tests differ among different clinical specimens, including oropharyngeal swabs, feces, urine, and blood [14]. Due to the limited results on consecutive sample collection of nucleic acids in clinical specimens [18], it is difficult to evaluate the persistence of the virus from the onset of the disease to the healing stage using different clinical specimens.

The high infectivity of 2019-nCov has been indicated by high control reproduction number and explosive growth of confirmed cases in China [3, 19]. Once the respiratory infection symptoms are manifested with suspicious contact history, the suspected individuals were isolated, protecting the susceptible population from the virus infection [20]. However, asymptomatic individuals carrying the virus exist, and they cannot be discovered until other individuals in close contact with the asymptomatic one become ill, potentially resulting in a cluster of infection or familial infection as reported [21]. Therefore, a detailed examination regarding the contacts that the infected people have had should be conducted in a community with cases of viral infection to isolate the virus carriers on time and prevent further transmission.

Our hospital was the only healthcare center for 2019-nCov treatment in Dongyang, a city of nearly 0.85 million people. An unknown pneumonia was first reported in Wuhan in December 2019, and approximately 7000 people who worked or studied in Wuhan visited Dongyang for the Spring Festival, bringing a threat to the local population. Therefore, this retrospective analysis on the transmission pathway and consecutive nucleic acid examination was performed to provide useful information for other regions to prevent further contagion and/or to be prepared to face another potential contagion of coronavirus in this region in the future.

## 2. Materials and Methods

The coronavirus-infected patients were diagnosed according to the guidelines for the diagnosis of 2019-nCov infection released by the National Health Commission. No identifiable information was displayed, and informed consent was obtained from all the patients on their admission to the hospital. All experimental protocols were approved by the Ethics Committee of Dongyang People's Hospital and Institutional Review Board.

**Figure 1:**
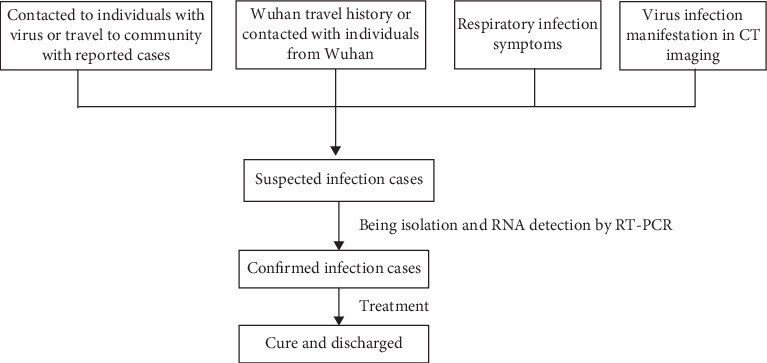
The main flow of diagnosis of 2019-nCov infection.

The main process of diagnosis is shown in Figure 1. Briefly, an individual was considered a suspected coronavirus patient based on the travel history and respiratory system infection. Next, he would be in isolation at the hospital and advised to perform a CT scan at a specific healthcare center. In addition, samples such as throat swabs, feces, sputum, and urine were collected and sent for RNA detection using RT-PCR. The total RNA was isolated using the RNA isolation kit provided by the manufacturers (Liferiver, China, http://www.liferiverbiotech.com/). The real-time RT-PCR was performed using the Applied Biosystems 7500 Real-Time PCR system according to the instructions from the manufacturers (Liferiver, China, RR-0479-02). The amplification conditions included pre-denaturation (at 45°C for 10 min and at 95°C for 3 min) and amplification (at 95°C for 15 sec and at 58°C for 30 sec) for 45 cycles. The lowest limit of detection was 1 × 10^3^ copies/ml.

If tested positive to the nucleic acid test, the patient was considered an infected case and received appropriate treatment. The information regarding the contacts that this infected patient had was collected, and the people with whom this patient had contacts were isolated within 14 days of contact. During the treatment in the hospital, continually collected samples were sent for the nucleic acid test and the potential pulmonary infection was also evaluated based on respiratory symptoms and CT imaging. The patients were discharged from the hospital only when results for RNA detection were negative two times, accompanied with the disappearance of the respiratory infection.

The significance between groups on continuous variables was analyzed using Student's *t* test. The difference between groups on categorical variables was tested by the *χ*^2^ test.

## 3. Results

The first case of 2019-nCov infection was reported on 22^nd^ January in Dongyang, and the recent one was identified on 16 February. All subsequently infected patients recovered by 1 March after various length of stay in the hospital. A total of 19 cases of 2019-nCov infection were confirmed in this region (Table 1). Among them, the infection progressed to a severe stage in 5 patients and four of them were transferred to the First Affiliated Hospital of Zhejiang University. The youngest patient was 13 years old, while the oldest one was 83; the average age was 46.1 years among the 19 patients. Six patients had travelled to or worked in Wuhan, and the remaining ones had a close contact with the other cases. In addition, 13 patients (68.4%) were subjected to hospitalization longer than two weeks.

**Figure 2:**
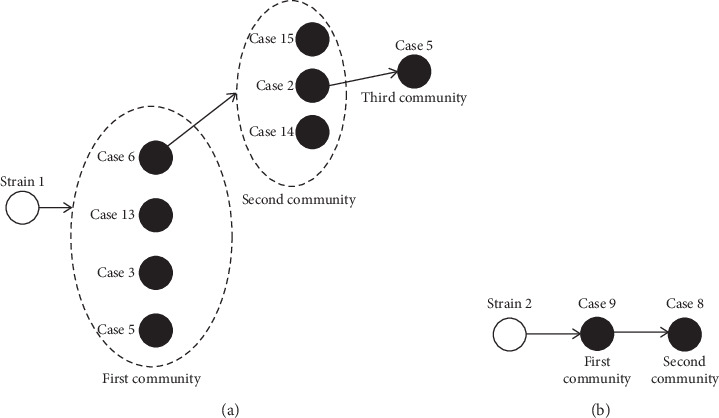
The transmission pathway of two virus strains leading to 10 clustered cases. (a) Strain 1 resulted in 8 cases. (b) Strain 2 resulted in 2 cases. The white circle refers to an asymptomatic individual with negative results in nucleic acid examination. The black circle refers to 2019-nCov-infected cases.

After tracing the people with whom these patients had contacts, it resulted that 11 cases were from two strains, while 8 cases occurred sporadically. A map is drawn to display a possible contact pathway in Figure 2. With regard to strain 1, four family members showed respiratory symptoms and then they were confirmed as 2019-nCov-infected cases through the nucleic acid test. They did not have a history of travelling to or from Wuhan or contact with the other suspected cases, but one of their family members who came from Wuhan was asymptomatic, with a pulmonary infection confirmed by the CT scan. This asymptomatic individual tested negative to the nucleic acid detection test, although three oropharyngeal swabs were collected and analyzed. Among the first four cases, the virus was transmitted from case 6 to a second community of patients, resulting in three cases (case 2, case 14, and case 15). Case 2 transmitted the virus to case 4. Strain 2 was also from an asymptomatic individual and transmitted to family members, resulting in one case.

The differences in clinical manifestations between clustered cases and sporadic cases are analyzed and shown in Table 2. The sporadic cases included a higher percentage of male patients and lower percentage of cases with severe stage, but without statistical significance compared to clustered cases. The mean age of the clustered cases was 54.9 years, which is higher than that of the sporadic cases (36.3 years) (54.9 ± 25.5 vs 36.3 ± 10.2, *P*=0.055). In addition, the clustered cases were likely characterized by a longer hospitalization compared to the sporadic cases (23.2 ± 5.9 vs 16.2 ± 9.8, *P*=0.088).

**Figure 3:**
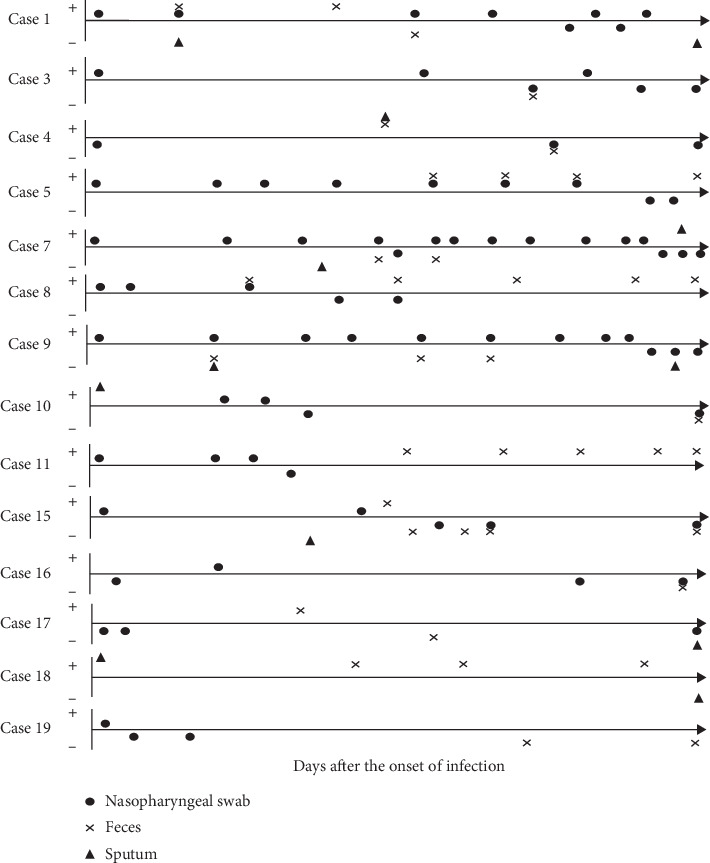
The successive nucleic acid examinations among different specimens from fourteen 2019-nCov-infected cases.

To analyze the conversion point in the nucleic acid results from different samples, the RNA results from the onset of the disease to discharge from the hospital are shown in Figure 3. Among these 14 analyzed cases with consecutive nucleic acid results (both negative and positive), the oropharyngeal swab was the most commonly tested sample type, followed by feces and sputum. As regards the samples collected at the same sampling time, 6 cases showed consistency between the results on feces and nasopharyngeal swabs, while 3 cases showed inconsistent results between different sample types. In addition, 4 cases tested positive for 2019-nCov in feces after their negative conversion in the oropharyngeal swab. From the onset of the disease till the recovery, positive results after a single negative result in the oropharyngeal swab were observed in five cases, while none converted from negative to positive in the feces samples.

## 4. Discussion

During the Spring Festival, nearly 7000 individuals from Wuhan travelled to Dongyang [22]. Fortunately, only 6 cases from Wuhan tested positive and 13 other cases were infected because they had contacts with the confirmed cases. The people with whom these patients had contact have been traced and their consecutive nucleic acid examination during hospitalization was performed and reported in this study.

The initial model predicting the transmission ability of 2019-nCov revealed that the virus has high infectivity, which is supported by our results that two strains caused 10 confirmed cases in our region [19]. When individuals with the virus could not be managed well, it resulted in clustered cases [21, 23, 24]. Without any intervention for prevention or control, the number of incidences would be much higher than that we detected. The accurate identification of individuals with the virus could be achieved by presenting a detailed history of the contacts they had, nucleic acid test, and even CT scan in highly suspected individuals [25]. Once the aforementioned history is complete, an essential intervention including isolation and diagnosis should be performed. Furthermore, the efficient control of transmission is largely dependent on the interventions of the governments, including any forbidden outdoor activity and strict self-isolation, which have been proven to be quite useful in preventing the explosive growth of cases [26, 27]. Daily fever surveillance in the community and nucleic acid testing among suspected individuals provide a timely diagnosis of 2019-nCov infection.

The existence of viral RNA in clinical specimens provide the clinicians sufficient evidence to diagnose 2019-nCov [13]. However, negative results have been reported in individuals who show typical pulmonary infection by CT imaging examination. The virus amount in clinical specimens varies along the process of the disease, showing fluctuations between negative and positive results in oropharyngeal swabs in our study. Delay in negative results in feces compared to that in oropharyngeal swabs during convalescence has been reported in a previous study, suggesting that nucleic acids in feces last longer than those in oropharyngeal swabs, which is also observed in our study [18]. Indeed, nucleic acids in feces or urine indicate that excrements from confirmed cases should be carefully managed. Virus could be spread through close contact and air-borne droplets from cough or sneeze, while other transmission routes such as fecal-oral transmission have not been confirmed [28]. However, nucleic acids in feces specimens last longer than those in oropharyngeal swabs in our study and others, indicating that fecal-oral route transmission should be reconsidered and reevaluated in a further study [18]. In this way, the diagnosis and discharge from the hospital based on nucleic acid detection may be modified according to different clinical specimens.

The limitation of this study is represented by the limited number of confirmed cases. However, the limited number of cases allows tracing them more easily and the transmission path between the cases can also be analyzed more easily. Findings of this study might help clinicians and researchers in evaluating the transmission ability of 2019-nCov and the appropriate sample to be used to assess the presence of viral nucleic acids.

## 5. Conclusions

Some of 2019-nCov strains could result in clustered cases and may be associated with longer hospitalization. Asymptomatic individuals could be identified via a detailed description of their contacts and should be treated with appropriate precautions, such as isolating in solitary confinement at the hospital or confined to the home. The viral nucleic acid test should also be performed in different types of clinical specimens.

## Figures and Tables

**Table 1:** Basic information of nineteen 2019-nCov-infected patients.

Features	Values	No. (%)

Gender	Female	7 (36.8)
	Male	12 (63.2)
Age (mean ± SD)	46.1 ± 21.5	
Clinical stage	Mild	14 (73.7)
	Severe	5 (26.3)
Contact history	Wuhan travel history	6 (31.6)
	Close contact with the infected cases	13 (68.4)
Symptoms	Fever	16 (84.2)
	Cough	9 (47.3)
	Pharyngalgia	2 (10.5)
	Muscle aches	4 (23.5)
	Nasal mucus	3 (21.1)
Coexisting diseases	Diabetes	1 (5.9)
	Hypertension	4 (23.5)
	Ankylosing spondylitis	2 (17.8)
Hospitalization duration (days)	≤14	6 (31.6)
	14∼28	10 (52.6)
	>28	3 (15.8)

**Table 2:** Comparison of clinical features between clustered patients and sporadic patients.

Features	Values	Clustered	Nonclustered	*P*

Gender	Male	6 (60)	6 (66.7)	1.0
	Female	4 (40)	3 (33.3)	
Age		54.9 ± 25.5	36.3 ± 10.2	0.0554
Clinical stage	Severe	4 (40)	1 (11.1)	0.303
	Mild	6 (60)	8 (88.9)	
Hospitalization duration (days)		23.2 ± 5.9	16.2 ± 9.8	0.0877

## Data Availability

The data used to support the findings of this study are included within the article.
